# Effects of Cold Rolling Reduction Rate on the Microstructure and Properties of Cu-1.16Ni-0.36Cr Alloy after Thermo-Mechanical Treatment

**DOI:** 10.3390/ma16196508

**Published:** 2023-09-30

**Authors:** Wenming Sun, Shaolin Li, Kexing Song, Qiangsong Wang, Yingying Zhu

**Affiliations:** 1 School of Material Science and Engineering, Henan University of Science and Technology, Luoyang 471023, China; swm2356320465@163.com (W.S.); yingzhu0425@163.com (Y.Z.); 2 Provincial and Ministerial Co-Construction Collaborative Innovation Center of Nonferrous New Materials and Advanced Processing Technology, Luoyang 471023, China; 3 Henan Academy of Sciences, Zhengzhou 450002, China; 4 State Key Laboratory of Nonferrous Metals and Processes, GRINM Group Co., Ltd., Beijing 100088, China; wangqiangsongbj@163.com; 5 GRIMAT Engineering Institute Co., Ltd., Beijing 101407, China

**Keywords:** Cu-Ni-Cr alloy, rolling strain, microstructure, strengthening mechanism

## Abstract

In this paper, a Cu-Ni-Cr alloy was prepared by adding a Ni-Cr intermediate alloy to copper. The effects of the cold rolling reduction rate on the microstructure and properties of the Cu-1.16Ni-0.36Cr alloy after thermo-mechanical treatment were studied. The results show that the tensile strength of the alloy increased while the electrical conductivity slightly decreased with an increase of the cold rolling reduction rate. At a rolling strain of 3.2, the tensile strength was 512.0 MPa and the conductivity was 45.5% IACS. At a rolling strain of 4.3, the strength further increased to 536.1 MPa and the conductivity decreased to 41.9% IACS. The grain size and dislocation density decreased with an increase of the reduction rate in the thermo-mechanical treatment. However, when the rolling strain reached 4.3, the recrystallization degree of the alloy increased due to an accumulation of the dislocation density and deformation energy, resulting in a slight increase in the grain size and a decrease in the dislocation density. The texture strength of the brass increased due to the induced shear band, with an increase of the cold rolling reduction rate. The reduction rate promoted a uniform distribution of nano-scale Cr precipitates and further enhanced the strength via precipitation strengthening.

## 1. Introduction

Copper alloys show potential excellent conductivity, mechanical properties, and formability, and are widely used in many high-tech fields, such as lead frames, connectors, asynchronous traction motors, aerospace, etc. [1,2]. In the research field of copper alloys, alloying is often performed by adding alloying elements to improve the mechanical properties and high-temperature properties of the alloy, and further improve the properties of the alloy through plastic deformation and heat treatment. Ni elements can be dissolved infinitely in a copper matrix, which is a commonly used solid solution strengthening element in copper alloys. However, the effects of solid solution strengthening are limited for copper alloys, and they have a great influence on the conduction properties. Cr elements are one of the most used alloying elements in precipitated strengthened copper alloy. By incorporating chromium into a copper alloy as a strengthening element for precipitation, the strength of the copper alloy can be enhanced through the formation of nano-scale precipitates during aging treatments. Additionally, this approach minimizes the impact on electrical conductivity [3]. In this paper, a Cr element is introduced by adding a Ni-Cr intermediate alloy. The Cr element is integrated into the copper matrix along with the Ni element, which promotes the uniform distribution of the Cr element in the matrix [4]. Therefore, the addition of Cr into the Cu-Ni system is anticipated to yield copper alloys that possess both high strength and electrical conductivity. The research on Cu-Ni-Cr alloys mainly focuses on phase diagram calculations, coarsening mechanisms, and composition designs [5,6,7]. Hernandez-Santiago et al. [8] analyzed the coarsening process of Cu-Ni-Cr alloy phases and found that with an increase of the aging time, the amplitude modulation structure was gradually replaced by the rectangular Ni-rich phase, which was coherently decomposed and finally became elliptic, and the addition of Cr enhanced the atomic diffusion in the alloy system. With an increase of the Cr content, the average radius of the Ni-rich phase and Cr precipitates increased at the same aging time. Li et al. [4] found that by controlling a certain nickel-chromium ratio and by changing the alloy content in the alloy system, an increase of the Cu content led to an increase of the cubic precipitates inside the alloy, which improved the strength of the alloy. The inclusion of higher amounts of Cr resulted in the formation of numerous Cr-rich precipitates along the grain boundaries, which had a negative impact on the mechanical properties of the alloy. In recent years, the research on low-solute Cu-Ni-Cr alloys in the field of electrical materials has gradually attracted wide attention. Trivedi et al. [9] used a Cu-Ni-Cr alloy as an electrode material for an electric joint, and the mechanical properties of the joint did not deteriorate after aging for 15 days at 723 K. Wang et al. [10] found that a Cu-0.27Cr-0.19Ni alloy obtained excellent stress relaxation resistance after an aging treatment, and the residual stress remained at 93.1% after 24 h at 194.7 °C. In general, the microstructure determines the final performance. For the Cu-Ni-Cr alloy, the existence form, dislocation/twin characteristics, grain size/orientation, and other microstructure characteristics of the alloying elements are complicated due to the presence of both Ni solution elements and Cr precipitates. The influence of mechanical heat treatments on the microstructure of the Cu-Ni-Cr alloy has not been extensively investigated. It is crucial to examine the evolution of the alloy’s microstructure in order to facilitate its design and application.

Thermo-mechanical treatment is an effective method to control the microstructure characteristics of copper alloys. The application of cold rolling treatments can effectively reduce the grain size of the alloy and enhance the density of dislocations, leading to a significant improvement in its strength. Subsequent aging treatments can promote the precipitation of the strengthened phase and effectively improve the strength and electrical conductivity of the alloy [11,12,13]. Fu et al. [11] showed that the tensile strength of a Cu-Cr-Zr alloy increased with the increase of the reduction rate during thermo-mechanical treatment. After an aging treatment, many Cr and Cu4Zr phases were precipitated, which greatly improved the conductivity of the alloy. Sun et al. [13] obtained a Cu-1Cr-1Ni-0.6Si alloy by adding Ni and Si to the Cu-Cr alloy as strengthening elements and then promoted the precipitation of the precipitates through a two-step thermo-mechanical treatment, which significantly enhanced the precipitation strengthening effect of the alloy and reduced the scattering of electrons. Wu et al. [14] found that a thermo-mechanical treatment significantly improved the mechanical properties of a Cu-1.5Ni-0.5Cr alloy, and the dispersion of the supersaturated Cr within the matrix was identified as the primary factor contributing to the enhancement of the alloy’s mechanical properties. The above studies show that thermo-mechanical treatment is an important means to control the microstructure and properties of the alloy, and the reduction rate is an important parameter in the cold rolling process, which directly affects the internal microstructure and precipitate behavior of the alloy. The Cu-Ni-Cr alloy exhibits excellent processability and can concurrently achieve substantial strengthening through cold deformation, in order to study the applicational value of the alloy as an electronic component. However, the influence of the reduction rate on the properties and microstructure evolution of the Cu-Ni-Cr alloy is rarely reported.

In this study, a thermo-mechanical treatment process was employed to control the microstructure and properties of the Cu-Ni-Cr alloy. Specifically, the influence of cold rolling on the microstructure and properties of the alloy was investigated. The evolution laws of various microstructural features, including grain size, texture, precipitation phases, and dislocations, were examined, along with their effects on the alloy’s properties. Furthermore, a quantitative analysis was conducted to determine the strengthening effect of each strengthening mechanism on the mechanical properties of the alloy.

## 2. Experimental Section

### 2.1. Materials and Preparation

**Figure 1:**
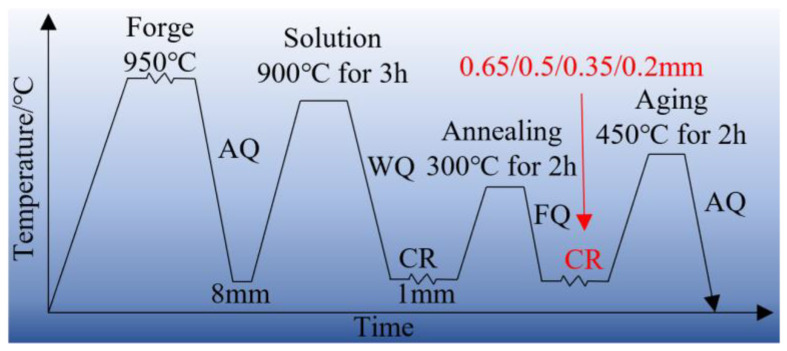
Schematic illustration of deformation and heat treatment process.

The cathode pure copper (99.99 wt%) and Ni-Cr intermediate alloys were melted in an intermediate frequency induction furnace, and charcoal was used as a cover agent for atmospheric melting, and they were then cast into a graphite crucible to obtain a cylindrical ingot. The ingot was homogenized at 950 °C for 12 h to eliminate the segregation of alloying elements. The actual composition of the alloy was determined to be Cu-1.16 wt% Ni-0.36 wt% Cr. Through previous work, the best hot working process of the alloy was 950 °C, 1 s^−1^, and the alloy was forged into a plate of 200 mm × 160 mm × 8 mm. Before cold rolling, the forged alloy was treated with a solid solution at 900 °C, with a solution time of 3 h, and the cooling medium was water. And then the alloy was cold rolled to 1 mm, and the rolling strain was 87.5%. The first cold-rolled alloy underwent an annealing process at 300 °C for 2 h. This annealing treatment was performed to partially eliminate the work hardening that occurred during cold rolling. The alloy was then cooled in a furnace to obtain the annealed alloy with reduced work hardening. The cold-rolling deformation of the second cold rolling was 35%, 50%, 65%, and 80%, and the thickness of the copper alloy sheet was 0.65 mm, 0.5 mm, 0.35 mm, and 0.2 mm, respectively. Subsequently, the copper alloy sheets with different thicknesses were aged at 450 °C for 2 h to obtain the aged alloy. The schematic illustration of the deformation and heat treatment process are shown in Figure 1. The rolling strain is described by the rolled strain ratio ƞ. $ƞ=2/\sqrt{3}ln\left(h_{0}/h\right)$, *h*: the uniform thickness of the plate cross-section after rolling; $h_{0}$: the initial uniform thickness of the plate cross-section. Copper alloy plates with specific thicknesses of 0.65 mm, 0.5 mm, 0.35 mm, and 0.2 mm were classified into a distinct rolling strain, namely 2.9, 3.2, 3.6, and 4.3, respectively.

### 2.2. Test Methods

The tensile tests were carried out for the alloy at room temperature by the WDW-100D precision universal material testing machine at a strain rate of 1.0 × 10^−3^ s^−1^. Dog-bone-shaped tensile samples with a width of 10 mm and a gauge length of 30 mm were processed by the national standard for GB/T 228.1–2010 [15]. The tensile test was carried out three times, and the data were verified statistically. The electrical conductivity at room temperature of the Cu-Ni-Cr thin alloy plate was measured by the Sigma 2008 B1 digital eddy current metal conductivity meter (⌀ 16 mm) (Sigma Aldrich, St. Louis, MO, USA). Each sample was measured at different positions five times to obtain the average value. The unit of electrical conductivity was %IACS. The micro-hardness of the alloy at room temperature was measured by the HVS-1000A digital tester with a 100 g load for 15 s.

The dislocation density was detected by a Bruker (Billerica, MA, USA) D8 X-ray diffractometer, with the scanning range varying from 40 to 100 deg at a step size of ~0.02 deg and a scanning velocity of ~4 deg/min. The microstructure of the alloy was analyzed by an inverted metallographic microscope (DMi8C, Leica Microsystems Inc., Wetzlar, Germany) and a scanning electron microscope (SEM, JSM-5610LV, JEOL, Tokyo, Japan). Electron backscattering diffraction (EBSD) was performed on the alloy in the elongation direction, and the sample information was collected by a JMS-7800F field emission scanning electron microscope. The acceleration voltage was 20 kV, the sample tilt was 70°, and the hit rate of each sample was guaranteed to be above 85%. The alloy was observed by JEM-2100 transmission electron microscopy (TEM), and the alloy was polished to 50–70 μm. Then, a 3 mm wafer was obtained by a punching machine, and the required samples were prepared by the Gatan659 particle thinning instrument (Gatan, Pleasanton, CA, USA).

## 3. Results

### 3.1. Effects of Reduction Rate on Properties of Alloy

**Figure 2:**
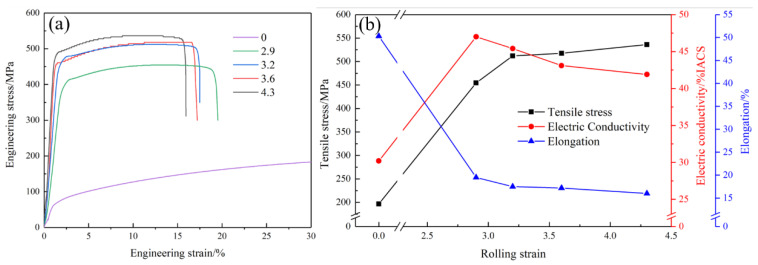
Curves of the Cu-Ni-Cr alloy at different rolling strains. (**a**) Strain–stress curve; (**b**) Change curve of tensile strength, elongation, and electrical conductivity.

Figure 2 shows the stress–strain curves and physical properties of the Cu-Ni-Cr alloy at different rolling strains. With an increase of the cold rolling reduction rate, the strength of the Cu-Ni-Cr alloy increases continuously. When the rolling strain reaches 2.9, the strength of the Cu-Ni-Cr alloy increases from 196.7 MPa to 454.5 MPa, and when the rolling strain rises to 4.3, the strength of the Cu-Ni-Cr alloy increases to 536.1 MPa, an increase of 18%. With an increase of the cold rolling reduction rate, the elongation decreases rapidly. When the alloy is in a solid solution state, the elongation is 49.7%, and when the rolling strain reaches 2.9, the elongation decreases to 19.5%. With a further increase of the cold rolling shape variable, the elongation shows little change. The conductivity of the solid solution alloy is 30.2% IACS. The electrical conductivity of the copper alloy significantly increases after undergoing the cold rolling and aging treatments, as compared to its initial solution state. When the rolling strain is 2.9, the electrical conductivity of the copper alloy reaches 47% IACS. However, as the reduction rate increases, the conductivity gradually decreases. When the rolling strain reaches 4.3, the conductivity of the alloy decreases to 41.9% IACS. This decrease in conductivity can be attributed to the higher reduction rate, which leads to the formation of internal defects such as dislocations and enhanced textures.

### 3.2. Effects of Reduction Rate on Microstructure of Alloy

**Figure 3:**
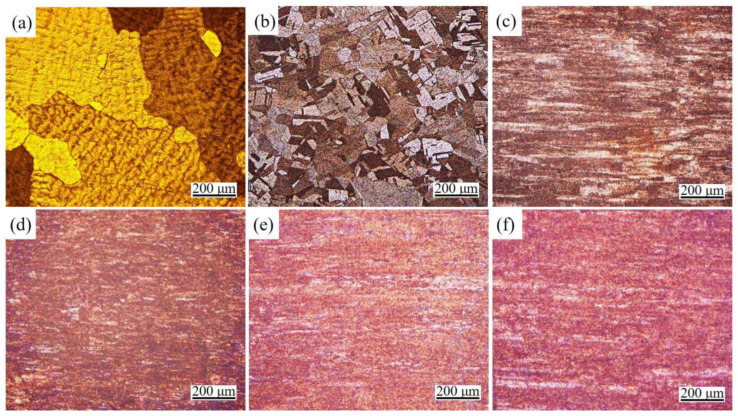
OM micrographs of the Cu-Ni-Cr alloy in different states: (**a**) As-cast; (**b**) Forging state; (**c**–**f**) Aging state with a rolling strain of 2.9, 3.2, 3.6, and 4.3.

Figure 3a displays the microstructure of the as-cast Cu-1.16Ni-0.36Cr alloy, showing coarse dendrites distributed within the grains. Figure 3b illustrates the microstructure of the alloy after forging and solution treatment. At this stage, the dendrites transform into fine and uniformly distributed equiaxed crystals. Additionally, a significant number of annealing twins form within the grains, resulting in a finer grain size. Figure 3c–f depict the OM images of the alloy under different cold rolling reduction rates. At this point, the original grain structure of the alloy becomes blurred, indicating a significant elongation of the grains. Simultaneously, the extent of the deformation increases with the rising rolling strain.

**Figure 4:**
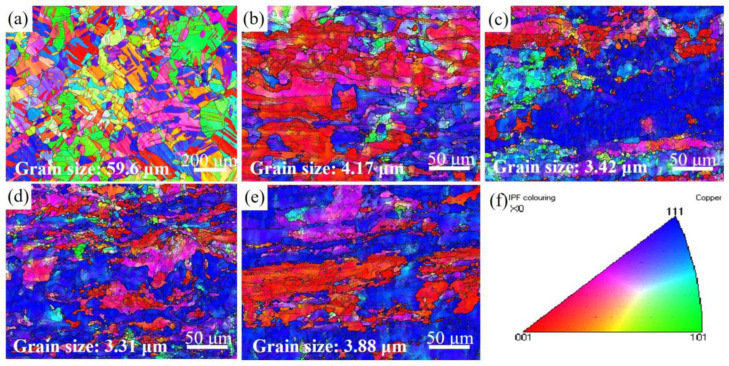
IPF maps of the Cu-Ni-Cr alloy in different states: (**a**) Solid solution state; (**b**–**e**) Aging state alloy with a rolling strain of 2.9, 3.2, 3.6, and 4.3; (**f**) ipf color.

Figure 4 shows the inverse pole figure (IPF) maps of the Cu-1.16Ni-0.36Cr alloy in the solution state and aging state under different rolling strains. In this figure, large angle grain boundaries with a grain boundary angle greater than 15° are marked with thick solid lines, and small angle grain boundaries with grain boundary angles ranging from 2° to 15° are marked with thin solid lines. The ipf map coloring is shown in Figure 4f. The solution-treated Cu-Ni-Cr alloy is composed of homogeneous equiaxed crystals with a large number of annealing twins, and the grain size is 59.6 μm. After the cold rolling treatment, the grains are composed of deformed grains and a lot of fine grains. In Figure 4b, when the rolling strain is 2.9, the grain size is greatly reduced to 4.17 μm, showing elongated grains. The results of ipf coloring show that a large number of grains are produced after the cold rolling aging treatment, which has an orientation of <001>ǁ ND (normal direction). At the same time, a large number of fine solid lines representing small angle grain boundaries are observed in the grain interior, which indicates that a large number of substructures are formed in the alloy during the cold rolling reduction rate. With an increase of the rolling strain, the grains in the <001> direction are gradually replaced by the grains in the <111> direction. When the rolling strain reaches 3.2, the grain size is further reduced to 3.42 μm. At this time, the grains are elongated and refined with the increase of the rolling strain. Fine equiaxed grains begin to appear around the large grains. As the rate of reduction continues to increase, when the rolling strain reaches 3.6, the grain size is 3.31 μm. As shown in Figure 4d, the elongated large grains are almost completely replaced by elongated fine grains and equiaxed grains. When the rolling strain reaches 4.3, the average grain size begins to increase to 3.88 μm, and there are a lot of elongated fine equiaxed grains and fewer substructures in the crystal. The results show that in the large strain zone, the preferred orientation of the grains in the alloy is more obvious, and it is difficult to refine the substructures through cold rolling. At this time, due to the increase in the reduction rate, the energy stored at the grain boundaries increases, so the degree of recrystallization increases after the aging treatment. When the rolling strain increases to 4.3, the grain elongation caused by cold rolling becomes dominant again. Currently, there are fine and uniform elongated grains in the alloy, which leads to a significant increase in strength and a decrease in plasticity.

**Figure 5:**
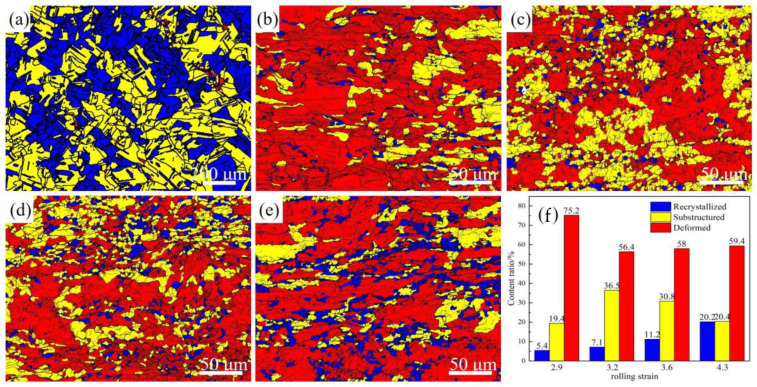
(**a**) Recrystallization degree diagram of solid solution alloy; (**b**–**e**) Recrystallization degree diagram of aging alloy with rolling strains of 2.9, 3.2, 3.6, and 4.3; (**f**) Recrystallization degree statistics.

Figure 5 shows the recrystallization degree of the alloy grains at different rolling strains. As shown in Figure 5a, after solution treatment of the Cu-Ni-Cr alloy, there are almost no deformed grains in the alloy. As shown in Figure 5f, with an increase of the rolling strain, the degree of recrystallization of the Cu-Ni-Cr alloy increases gradually after the aging treatment, and when the rolling strain reaches 4.3, the degree of recrystallization reaches 20.4%. It can be seen from Figure 5b–e that when the rolling strain is 2.9, there are almost all deformed grains in the alloy, only a few recrystallized grains, and the grains are obviously stretched. When the rolling strain reaches 3.2, the grain size decreases obviously, and many sub-crystals appear. At the same time, fine and uniform recrystallized grains appear at grain boundaries. When the rolling strain further increases to 3.6, the grains become finer, and a large number of fine and uniform recrystallized grains begin to appear. In this case, the alloy is composed of large, deformed grains and a large number of fine sub-crystals and equiaxed grains. When the strain level further reaches 4.3, the grains show obvious directionality and a large number of fine equiaxed crystals appear at the grain boundaries. Meanwhile, compared with the recrystallized grains in Figure 5d, the recrystallized grains grow significantly, indicating that the recrystallization is promoted by the increase of the alloy deformation and intergranular energy storage.

### 3.3. Effects of Cold Rolling Strain on Dislocation Density of Aging Alloy

**Figure 6:**
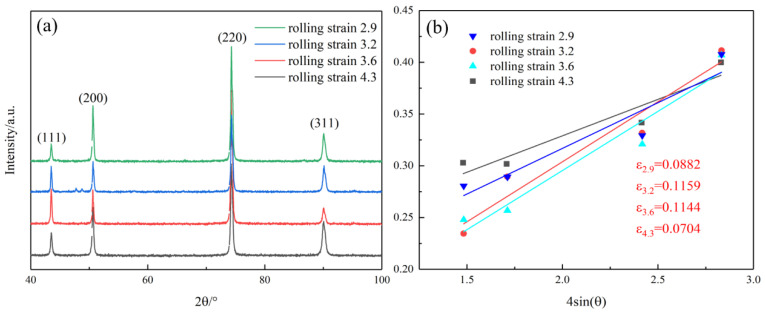
(**a**) XRD patterns of aged Cu-Ni-Cr alloys at different rolling strains; (**b**) Linear fitting plot of the alloy at different rolling strains.

The XRD images of the alloys at different rolling strains are shown in Figure 6a. With an increase of the rolling strain, the strength of the <111> peak increases, which is similar to the result of EBSD. The XRD patterns show that there is no obvious peak shift in the cold rolling process. However, with the increase of the cold rolling strain, the half-peak width changes, which shows a change of the micro-strain in the alloy after the thermo-mechanical treatment. According to the Williamson–Hall equation [16], the micro-strain in the alloy can be reflected by X-ray diffraction data.

(1) $$\beta \cos\left(\theta \right)=\frac{k\lambda }{d}+4\varepsilon \sin(\theta )$$

where β is the half-peak width of the maximum diffraction peak, λ is the radiation wavelength, *k* is a constant of 0.9, θ is the Bragg Angle, and d is the grain size. Figure 6b shows the 4sin(θ)-$\beta \cos\left(\theta \right)$ images of the alloy at different rolling strains. According to Equation (1), the linear fitting of the experimental data shows that the slopes are 0.0882, 0.1159, 0.1144, and 0.0704, respectively.

Therefore, the dislocation density of the alloy can be calculated from the total micro-strain ε [17].

(2) $$\rho =\frac{2\sqrt{3}\varepsilon }{db}$$

where b is the Burgers vector equal to $\sqrt{2}a/2$, take 0.255 nm, *a* is the lattice constant of the copper alloy [18], and *d* is the grain diameter. According to EBSD, the average grain diameter of the alloy with the rolling strain of 2.9, 3.2, 3.6, and 4.3 is 4.17 μm, 3.42 μm, 3.31 μm, and 3.88 μm, respectively. Therefore, the dislocation densities under different alloy states are calculated as 2.87 × $10^{14}{/m}^{2}$, 4.61 × $10^{14}{/m}^{2}$, 4.69 × $10^{14}{/m}^{2}$, and 2.47 × $10^{14}{/m}^{2}$, respectively. The results show that the internal dislocation of the alloy increases rapidly with an increase of the rolling strain and becomes stable when it reaches 3.2. As the rolling strain continues to rise to 4.3, the dislocation density decreases due to the increased degree of recrystallization and the rapid reduction of the substructure in the alloy, as shown in Figure 4.

### 3.4. Effects of Reduction Rate on the Texture of the Alloy

**Figure 7:**
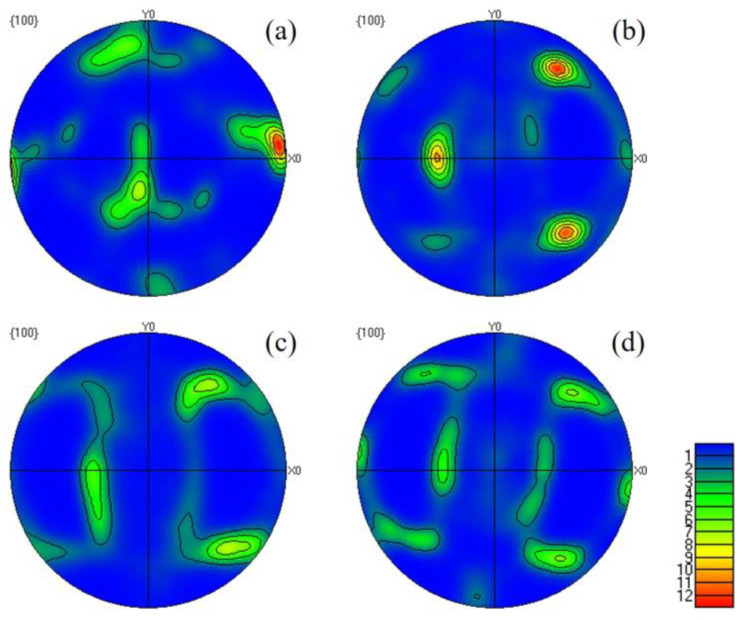
(**a**–**d**) Pole figure of aging alloy with a rolling strain of 2.9, 3.2, 3.6, and 4.3.

Figure 7 presents the pole diagram of the alloy in the 001 direction under various rolling strains. This diagram indicates that at strain levels of 2.9, 3.2, 3.6, and 4.3, the maximum strength of the internal texture of the alloy is measured as 12.58, 11.28, 9.84, and 8.64, respectively. Based on Figure 7a, it is evident that when the rolling strain is 2.9, the texture is concentrated in the X0 direction, indicating that the grains are elongated along the rolling direction. The texture is mainly an S texture, which corresponds to a large number of grains parallel to the <100> direction in Figure 4b. With an increasing strain, the maximum strength of the texture gradually decreases, and the texture aligned with the rolling direction transitions towards the <110> and <111> directions. With further increases in the rolling strain, the textures in the <110> and <111> directions stabilize. It can be inferred that the change in texture, with the increasing rolling strain, represents a transformation from the S texture to the Brass texture {011} <211> and the copper texture {112} <111>.

**Figure 8:**
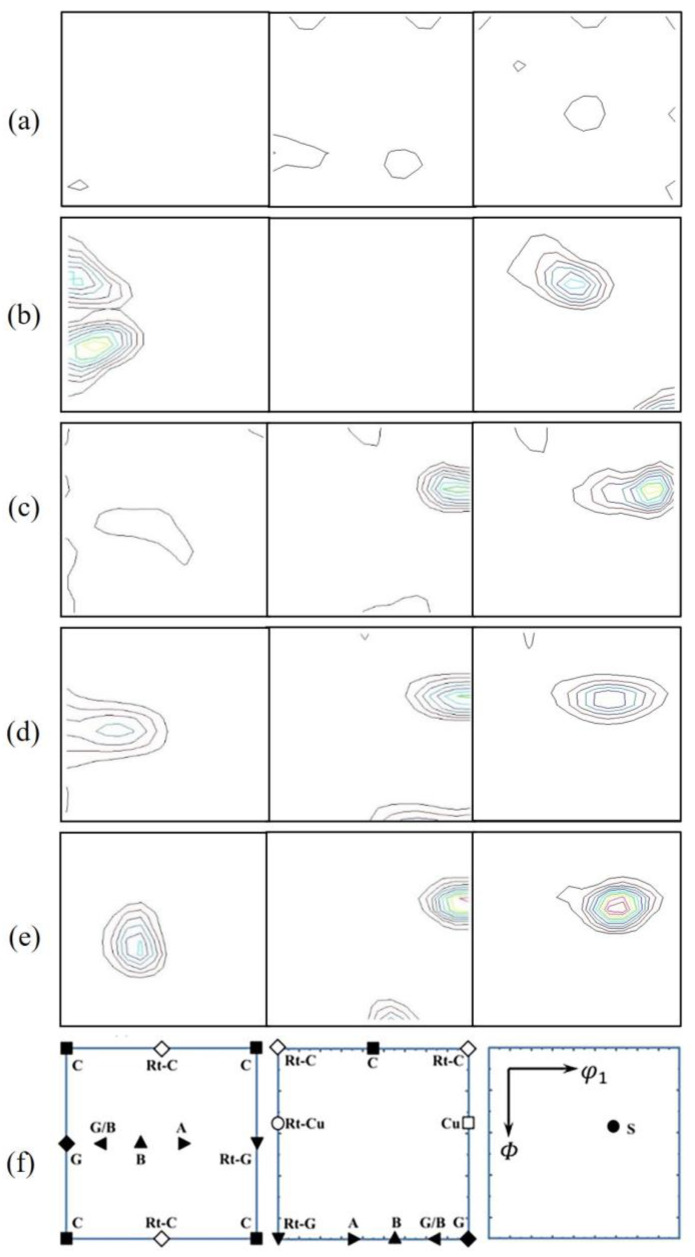
(**a**–**e**) ODF diagram of the aging alloy at 0°, 45°, and 65° with a solid solution state and rolling strain of 2.9, 3.2, 3.6, and 4.3; (**f**) Locational map of typical texture distribution of FCC alloy.

For FCC materials, there are mainly two basic textures: copper texture and brass texture [19,20]. The ODF diagram of the Cu-Ni-Cr alloy at 0°, 45°, and 65° sections under different rolling strains is shown in Figure 8, and the typical preferential orientation of the rolled FCC metal is shown in Figure 8f [11]. The evolution of the internal texture of the alloy with an increase in the reduction rate can be obtained from the ODF diagram. In Figure 8a, after solution treatment, the texture strength of the Cu-Ni-Cr alloy is weak, and the texture distribution is almost random. After cold rolling, as shown in Figure 8b, a strong S texture {123} <634> is formed; the copper texture and brass texture were generated when the rolling strain reached 3.2. The copper texture increases rapidly to a higher degree and the texture of brass increases slowly. When the rolling strain reached 4.3, the copper texture, brass texture, and S texture all reached their maximum values, and the texture density significantly increased.

The texture evolution of the alloy during cold rolling is mainly manifested by the change of orientation from the <001> orientation to the <111> orientation. The strength of the brass texture increases with an increase of the cold rolling reduction rate. The strength of the brass texture remains at a low level when the rolling strain is 3.2. When the rolling strain continues to increase, the texture strength of the brass increases rapidly, which indicates that when the rolling strain is high, the deformation of the alloy is hindered. The deformation mode is mainly shear deformation, and shear deformation promotes the development of the brass texture [21]. The strength of the copper texture increased with an increase of the cold rolling reduction rate. It increases rapidly in the early strain stage and tends to be stable in the late strain stage. In the process of cold rolling, the sliding deformation is the main deformation at the initial stage of the strain, and with an increase of the cold rolling duction rate, the internal deformation of the alloy is mainly shear deformation. In copper-based alloys, the <111> orientation of the copper texture has the least slip system and lower Schmidt factor compared with the <001> and <011> orientations, so it is the hardest orientation to deform [22]. It was shown that when the rolling strain reached the maximum of 4.3, the strength of the copper texture reached the maximum, which revealed that the strength of the rolled alloy reached the maximum at this rolling strain and that the elongation was low.

### 3.5. Microstructure Evolution of Alloys

**Figure 9:**
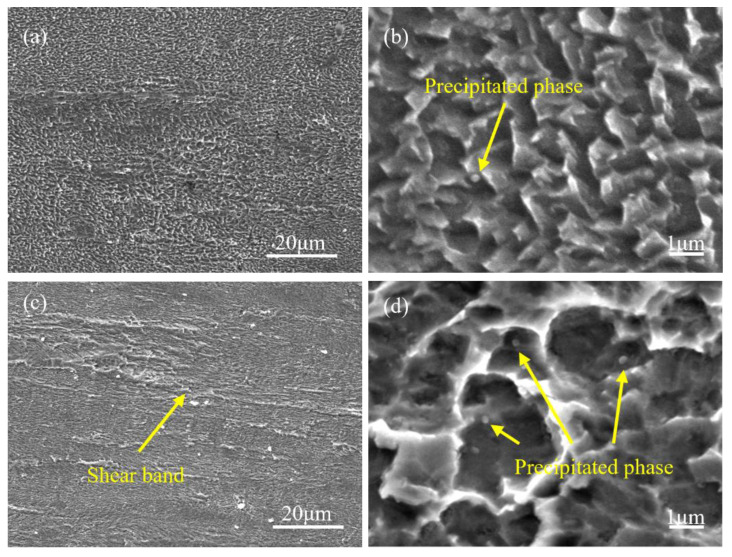
SEM of alloy at different rolling strains (**a**,**b**) 3.2; (**c**,**d**) 4.3.

To further study the effects of the thermo-mechanical treatment process on the internal microstructure deformation and aging precipitation behavior of the Cu-Ni-Cr alloy, the microstructure of the solid solution state and aging state alloy was observed by SEM and TEM. Figure 9 shows the microstructure of the aging alloy at different rolling strains observed by SEM. After the cold rolling and aging treatments of the alloy, when the rolling strain is 3.2, the grain is obviously compressed and elongated, as shown in Figure 9a. With an increase of the reduction rate, when the reduction rate reaches 4.3, obvious shear bands appear parallel to the rolling direction, as shown in Figure 9c, and an obvious directionality of the grains appears. In Figure 9b,d, there are sparse white precipitates on the grain surface with a uniform distribution. In Figure 9d, many small precipitates are uniformly distributed in the shear zone. Compared with the precipitates in Figure 9b, the precipitates are more densely distributed in the shear zone. In conclusion, the cold rolling reduction rate has a significant effect on the microstructure and precipitation behavior of the Cu-Ni-Cr aged alloy. Due to the large deformation in the cold rolling process, a fiber structure and shear band formed in the alloy, and these structures could be used as nucleation points to promote the generation of the second phase precipitation. With an increase of the cold rolling reduction rate, the shear band in the alloy increases, which further promotes the precipitates.

**Figure 10:**
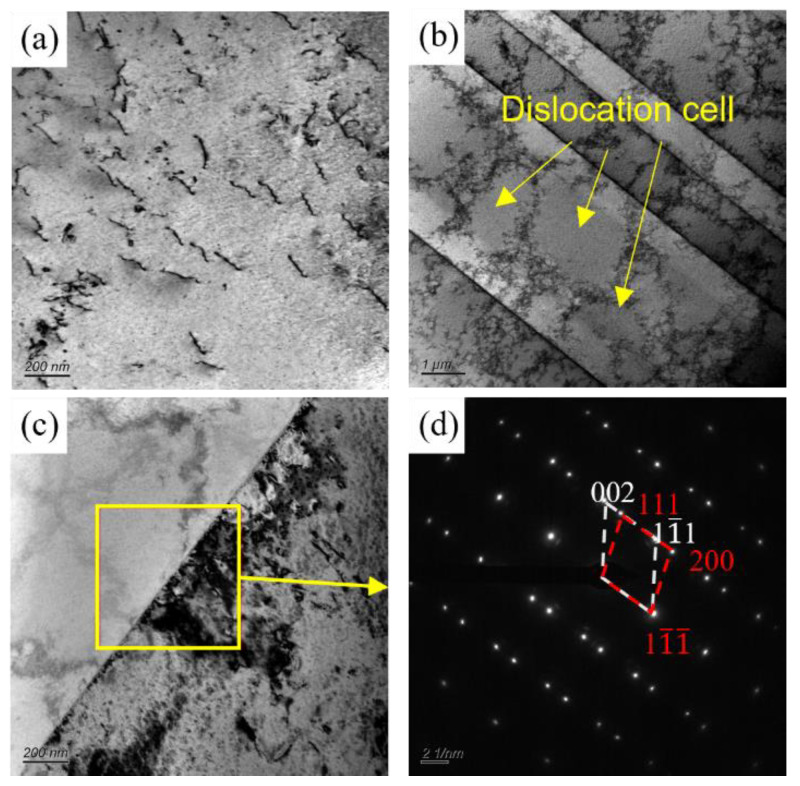
TEM micrographs of the Cu-Ni-Cr alloy in solid solution. (**a**) Bright field image of dislocation; (**b**) Bright field image of the twin; (**c**) Bright field image of twin boundary; (**d**) SAED image of the selected area in Figure 10c.

The microstructure of the Cu-Ni-Cr alloy was observed by TEM in order to investigate the effects of the cold rolling reduction rate and aging process on the microstructure and precipitation behavior of the Cu-Ni-Cr alloy. Figure 10 shows the TEM microstructure of the Cu-Ni-Cr alloy in a solid solution. As shown in Figure 10a, there are almost no other defects in the grain of the solid-solution alloy except that a few linear dislocations are observed to be uniformly and diffusely distributed on the copper matrix. In Figure 10b, many annealed twins are observed in the matrix, which is consistent with that observed by EBSD, with a width of 4 μm. According to the grain growth theory, the alloy underwent complete recrystallization after solution treatment. During the recrystallization process, the grain grows through grain boundary migration, which produces twin interfaces on the dense lattice surface. The face-centered cubic alloy has a low layer fault energy and is more prone to annealing twinning [23]. At the same time, there are a lot of dislocations in the twin, dislocation entanglement occurs, and dislocation cells are observed where the dislocations gather. The presence of a dislocation cell in the twin is observed.

**Figure 11:**
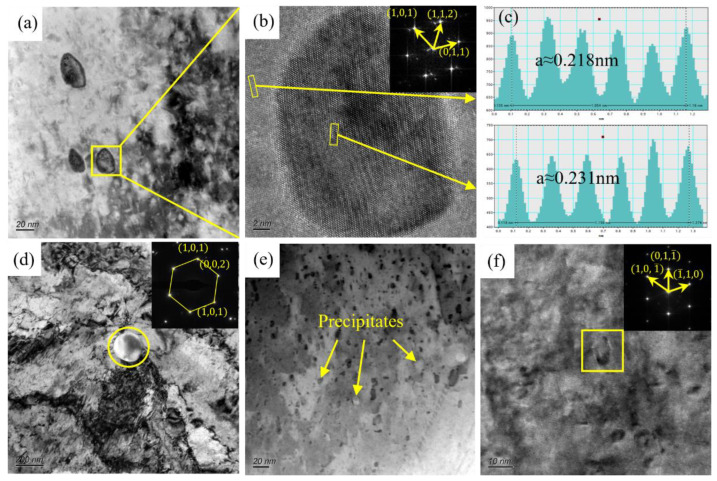
TEM microstructure of the aged Cu-Ni-Cr alloy with a rolling strain of 3.2: (**a**) Bright field phase of dislocation and precipitates; (**b**) HRTEM of yellow square in (**a**) and FFT of (**b**); (**c**) Crystal plane spacing of Cu matrix and Cr phase; (**d**) Bright field phase of dislocation and precipitates and FFT of precipitates in (**d**); (**e**) Bright field phase of nano-scale precipitates; (**f**) HRTEM of nano-scale precipitates and FFT of precipitates in (**f**).

Figure 11 shows the TEM micrographs of the aging Cu-Ni-Cr alloy with a rolling strain of 3.2. As shown in Figure 11a,d, two nano-scale precipitates are precipitated in the alloy when the rolling strain reaches 3.2 after thermo-mechanical treatment. One is the nano-scale precipitate with a size of less than 20 nm, and there are a lot of pod-like structures around the nano-scale precipitate, which means there are a lot of finer nano-scale precipitates near this type of precipitate. The other is the nano-scale precipitate with a size of about 100 nm, which exists in a small number and alone. SAED (Selected Area Electron Diffraction) was performed on the precipitates to obtain the Cr phase with a BCC (Body-Centered Cubic) structure. Figure 11b shows the HRTEM (High Resolution Transmission Electron Microscope) microstructure of the nano-scale precipitates, and the FFT (Fast Fourier Transform) results show that the precipitates are Cr phase with a BCC structure. The regular granular small-sized precipitated phase exhibits a stable structure, suggesting that the precipitation efficiency of the Cr precipitated phase is enhanced under significant deformation. Furthermore, the aging treatment results in the formation of a stable BCC structure, which is consistent with the structure of the larger-sized Cr particles. This simple precipitated phase is resistant to cutting and deformation. The interplanar spacing of the Cr phase and Cu matrix are 0.231 nm and 0.218 nm, respectively. Figure 10b shows that many dislocations are generated in the alloy, and the interaction between the dislocations and the micron phase is more intense than that of the annealed alloy. The relationship between the dislocations and precipitates is mainly based on the “bypass mechanism”. According to the interface mismatch formula [24], the mismatch between the precipitates and the matrix is 5.8%, and the relationship between the second phase and the matrix is semi-coherent.

(3) $$\delta =\frac{2(\alpha_{\beta }-\alpha_{\alpha })}{\alpha_{\beta }+\alpha_{\alpha }}\times 100\%$$

where $\alpha_{\beta }$ and $\alpha_{\alpha }$ are the surface distances of the two parallel interfaces of the secondary phase and the matrix, respectively. In Figure 11e, many nano-scale precipitates are dispersed in the alloy. The existence of grainy nano-scale precipitates was observed by HRTEM, and the FFT results show that the Cr phase with a BCC structure existed in this region. The results show that there are precipitates with the size of 3~5 nm in the alloy, which corresponds to the situation in Figure 11a. According to the statistics, the average size of the precipitates in the alloy is 4.8 nm when the rolling strain is 3.2.

**Figure 12:**
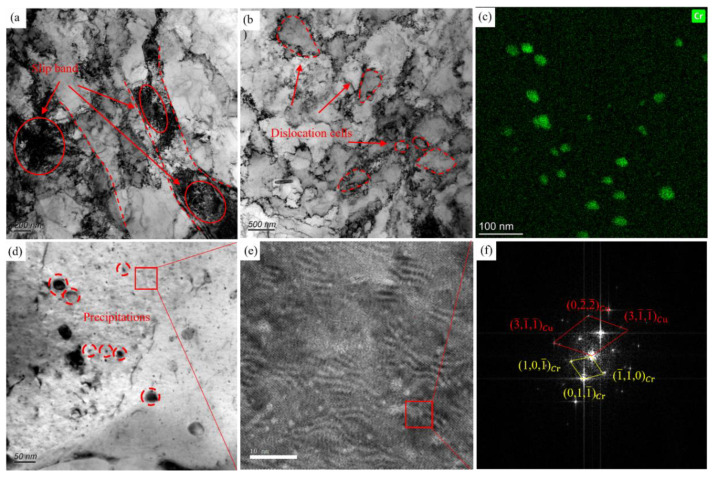
TEM microstructure of the aged Cu-Ni-Cr alloy with a rolling strain of 4.3: (**a**,**b**) Bright field phase of dislocation and dislocation cells; (**c**) EDS of (**d**); (**d**) Bright field phase of precipitates; (**e**) HRTEM of red square in (**b**); (**f**) FFT of red square in (**e**).

When the rolling strain of cold rolling is further increased to 4.3, more dislocation cells are found in the matrix, and the dislocation cells produced are smaller. When the cold rolling strain is further increased to 4.3, the accumulation of the alloy reduction rate increases, the sliding deformation is difficult, and a lot of shear deformation occurs in the grain. After the aging treatment, the degree of recrystallization increased. In Figure 12a, it is observed that the matrix structure elongates along the direction of the deformation, and obvious shear bands are observed in which a large number of dislocation tangles exist. With a further increase of the reduction rate, the driving force for recrystallization of the deformed grains increases. Many dislocation cells are observed in Figure 12b, which indicates that the aging treatment after the large deformation treatment generates more driving force for the recrystallization of the alloy. After cold rolling with large deformation, the dislocations in the alloy increase and promote the generation of precipitates. In Figure 12d, many fine and uniform nano-scale precipitates are observed. By surface scanning of the area in Figure 12d, it is found that the precipitates with larger sizes are Cr precipitates. The HRTEM microstructure of the regions with a large number of nano-scale precipitates was observed, and it was found that a large number of pod-like precipitates existed. The FFT revealed that the Cr phase with a BCC structure was in a semi-coherent relation with the copper matrix. The dispersion and uniform distribution of nano-scale precipitates in the alloy plays an important role in improving the strength of the alloy. According to the statistics of the size of nano-scale precipitates, the average size of the precipitates inside the alloy is 4.4 nm when the rolling strain is 4.3.

## 4. Discussion

### 4.1. Volume Fraction of Precipitates

The solute elements and precipitates in copper alloys play an important role in the properties of copper alloys. In order to analyze the precipitation process of solute atoms in the Cu-Ni-Cr alloy after thermo-mechanical treatment, the kinetics of precipitation during aging was described by Avrami’s law [25].

(4) $$\varphi =1-e^{-bt^{n}}$$

where φ is the volume fraction of precipitation, which is the ratio between the volume fraction of precipitation and the maximum volume fraction of precipitation under a certain aging time, *t* is the aging time, and *b* and *n* are constants. Take the logarithm of both sides of this equation.

(5) $$lg\left(ln\frac{1}{1-\varphi }\right)=lgb+nlgt$$

The scattering of electrons by solute atoms in solid solutions has a significant influence on the electrical conductivity of the alloy. In this study, it can be reasonably assumed that the electrical conductivity of the alloy is mainly affected by solute atoms other than the matrix [26]. As the aging time increases, the solute atoms are precipitated continuously, which weakens the influence of the solute atoms on the electrical conductivity, and the electrical conductivity of the alloy increases with the aging time. Therefore, the ratio between the residual content of the alloying elements in the matrix and precipitates can be calculated according to the conductivity of the alloy in different states, and the relationship between the conductivity of the alloy and the volume fraction of the precipitate in the alloy can be regarded as linear [27].

(6) $$\varphi =\frac{\xi -\xi_{0}}{\xi_{max}-\xi_{0}}$$

where ξ is the electrical conductivity of the Cu-Ni-Cr alloy at aging time t, $\xi_{0}$ is the electrical conductivity of the solid-solution alloy with a measured value of 30.2% IACS, and $\xi_{max}$ is the maximum electrical conductivity of the annealed alloy with a measured value of 48% IACS.

**Figure 13:**
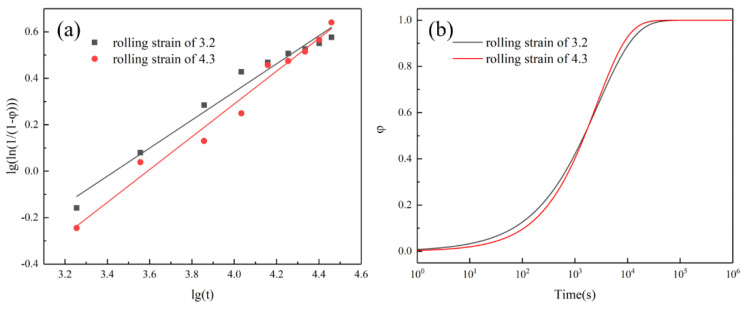
(**a**) $g\left(ln\frac{1}{1-\varphi }\right)$-lgt linear fit plot; (**b**) Precipitation volume versus time curve.

The electrical conductivity of the alloy with the rolling strains of 3.2 and 4.3 was measured at different aging times, and the corresponding φ value was calculated. The scatter plot of $lg\left(ln\frac{1}{1-\varphi }\right)$-lgt was drawn, and linear fitting of the data was performed according to Equation (5), as shown in Figure 13a. The values of *n* and *b* are measured from the slopes and intercepts of the fitted curves. Substituting the obtained values of *n* and *b* into Equation (5), the equation of the precipitation volume fraction evolving with the aging time can be obtained, and the curve obtained is shown in Figure 13b.

The copper-nickel binary system is considered to be an ideal isomorphous system. The Ni element can be dissolved indefinitely in the copper matrix, and the solution of Cr at 450 °C is almost zero. At the same time, due to the short aging time, it is considered that only Cr precipitates in the whole system. Figure 13b shows that after 2 h of aging, the value of φ is infinitely close to 1, indicating that the precipitates are completely precipitated under this condition. According to Figure 13b, after 2 h of aging, when the rolling strains are 3.2 and 4.3, the value of φ is infinitely close to 1, suggesting that the precipitates are completely precipitated at this time and the rolling strains have little influence on the precipitation volume. Next, we will calculate the volume fraction of the precipitates through Equation (7) [28].

(7) $$f=\frac{\sum \frac{m_{p}}{\rho_{p}}}{\frac{m_{Cu}}{\rho_{Cu}}+\sum \frac{m_{p}}{\rho_{p}}}$$

where $m_{p}$ is the mass when the precipitates are fully precipitated, $m_{Cu}$ is the mass of the copper matrix, $\rho_{p}$ is the density of the precipitates, $\rho_{Cr}=7.19$ g/cm^3^, and $\rho_{Cu}=8.9$ g/cm^3^. According to Figure 13b, after 2 h of aging, under the condition of rolling strains of 3.2 and 4.3, the values of φ are 83.5% and 84.4%. The corresponding volume fraction is 0.37% and 0.39%.

### 4.2. Strengthening Mechanism of the Cu-Ni-Cr Alloy

The mechanical properties of the Cu-Ni-Cr alloy plates are significantly enhanced after thermo-mechanical treatment, which is mainly related to the precipitation phase and grain evolution during cold rolling and heat treatment. The influence of various processes during thermo-mechanical treatment on the Cu-Ni-Cr alloy can be analyzed by studying the contribution of different strengthening mechanisms to the mechanical properties of the alloy. Four different strengthening mechanisms are discussed in this paper, including solution strengthening, precipitation strengthening, grain boundary strengthening, and dislocation strengthening.

After the aging treatment, Ni atoms are dissolved in the Cu matrix. The mismatch of the atomic size between the solute atoms and solvent atoms causes lattice distortion, which hinders the movement of dislocations and results in solution strengthening. In this paper, the Labusch theory [29] was used to quantitatively analyze the solution strengthening of the aged Cu-Ni-Cr alloy.

(8) $$∆\sigma_{ss}=MG_{0}{\left[{\left(\beta \frac{a_{1}-a_{0}}{a_{0}}\right)}^{2}+{\left(2\frac{G_{1}-G_{0}}{G_{1}+G_{0}}\right)}^{2}\right]}^{2/3}c^{2/3}$$

where *M* is the Taylor factor equal to 3.06 [30], *G*_0_ is the shear modulus of the copper matrix equal to 47 GPa, *a*_0_ is the lattice constant of the copper matrix equal to 0.361 nm, a_1_ is the lattice constant of the solute, Ni is 0.352 nm, and Cr is 0.2884 nm. *G*_1_ is the shear modulus of the solute atom, Ni is 77 GPa, and Cr is 115 GPa. *c* is the concentration of the solute atoms in the alloy [31,32]. In this alloy, Ni is almost completely dissolved in the matrix, and its solid solubility is considered to be 1.16%. The solid solubility of the Cr in the matrix varies with the stress variables. According to Figure 13, when the rolling strains are 3.2 and 4.3, the φ values are 0.37% and 0.39% and the c values are 0.059% and 0.045%, respectively. At this time, the contribution ∆σss of solid solution strengthening is 9.02 MPa and 8.67 MPa.

Dislocation strengthening means that in the process of introducing plastic deformation, the higher the dislocation density, the stronger the hindering effect of the dislocation movement and the stronger the ability of the alloy to resist deformation so that the mechanical properties of the alloy are enhanced. The contribution of dislocations to the strength of the alloy can be estimated by the Bailey–Hirsch formula [33].

(9) $$∆\sigma_{d}=M\alpha Gb\rho^{1/2}$$

where *M* is the Taylor factor, α is the constant of the copper alloy equal to 0.2, *G* is the shear modulus of the copper matrix, *b* is the Burgers vector, and ρ is the dislocation density. The dislocation densities of the alloy at rolling strains of 3.2 and 4.3 are obtained in Equation (2), which are 4.61 × $10^{14}{/m}^{2}$ and 2.47 × $10^{14}{/m}^{2}$. According to Equation (9), the $∆\sigma_{d}$ of the alloy with rolling strains of 3.2 and 4.3 are 157.5 MPa and 115.3 MPa, respectively.

Grain boundaries strengthen the material by impeding dislocation movements. Grain boundary strengthening can be calculated by the classical Hall–Petch formula [34].

(10) $$∆\sigma_{GB}=кd^{1/2}$$

к is the Hall–Petch coefficient, whereas the value к is different for the different materials and is 140 MPa ${\mu m}^{1/2}$ in the copper alloy. The average grain diameter of the alloy with rolling strains of 3.2 and 4.3 is 3.416 μm and 3.877 μm, respectively. Therefore, the contribution of grain boundary strengthening in the two states can be calculated as 75.8 MPa and 71.1 MPa, respectively.

The precipitation has a significant effect on the strength of the alloy. During the deformation process, the deformation of the alloy is limited by Cr particles, resulting in dislocation accumulation, which hinders the deformation of the alloy and improves its strength of the alloy. In the Cu-Ni-Cr alloy system, there are both 10 nm Cr particles and 100 nm larger Cr particles. However, the content of the Cr precipitates with large sizes is very small in the alloy, and it has little effect on precipitation strengthening [35]. With the aging process, the Cr precipitates gradually grow up and lose their coherent relationship with the matrix, which makes it difficult to shear Cr particles. The dispersed dispersion phase improves the alloy strength through pinning. Therefore, in this paper, the contribution of precipitation strengthening to the strength is considered as Orowan mechanism [36,37,38].

(11) $$∆\sigma_{p}=0.81\frac{MGb}{2\pi \sqrt{1-\upsilon }}\frac{ln(\bar{d}/b)}{\left(\lambda -\bar{d}\right)}$$

(12) $$\lambda =2r(\sqrt{\frac{\pi }{4f}}-1)$$

where *M* is the Taylor factor, υ is Poisson’s ratio of the copper matrix equal to 0.34, *G* is the shear modulus of the matrix, *b* is burgers vector, $\bar{r}$ is the average radius of the precipitates, $\bar{r}=\sqrt{2/3}r$, $\bar{d}$ is the average diameter of the precipitates, and λ is the distance between the precipitated edges, which can be obtained by Equation (12), where *f* is the volume fraction of the precipitates, which is 0.37% and 0.39% when the rolling strains are 3.2 and 4.3.

When the rolling strain of the Cu-Ni-Cr alloy reaches 3.2 after the cold rolling and aging processes, the average diameter and volume fraction of the precipitates in the alloy are 4.8 nm and 0.37% and the strength contributed by the Orowan mechanism is 144.9 MPa. When the rolling strain reaches 4.3, the average diameter of the precipitates is 4.2 nm, the volume fraction is 0.39%, and the strength contributed by the Orowan mechanism is 177.1 MPa. The strengthening effect of the alloy by cold rolling with large deformation is remarkable.

**Figure 14:**
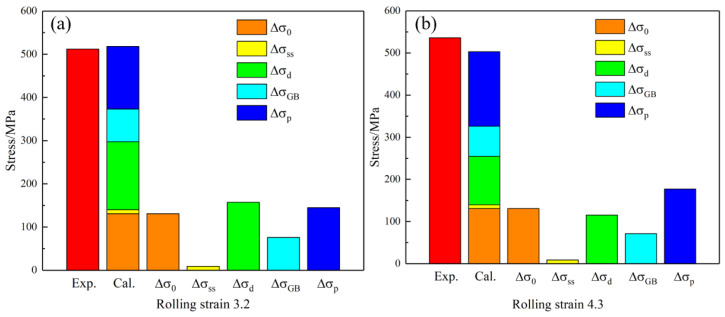
Contribution of strengthening mechanism to the strength of the Cu-Ni-Cr alloy under different rolling strains (**a**) 3.2; (**b**) 4.3.

The improvement of the Cu-Ni-Cr alloy’s strength can be considered as a superposition of the contribution of the four strengthening mechanisms to the strength: solution strengthening, dislocation strengthening, grain boundary strengthening, and precipitation strengthening. This can be expressed as the following equation:

(13) $$\Delta \sigma_{alloy}=\sigma_{0}+\Delta \sigma_{ss}+\Delta \sigma_{d}+\Delta \sigma_{GB}+\Delta \sigma_{p}$$

where $\Delta \sigma_{alloy}$ is the strength of the aging Cu-Ni-Cr alloy and $\sigma_{0}$ is the strength of the copper matrix. The strength of the copper matrix is 131 MPa by a tensile test of the pure copper as cast. According to the above analysis, the contribution of each strengthening mechanism to the strength of the Cu-Ni-Cr alloy is shown in Figure 14. The calculated results are larger than the test results, mainly because there are two sizes of the Cr precipitated phase, and the large sized precipitated phase relatively reduces the precipitation strengthening of the alloy.

According to the contribution of each strengthening mechanism to the strength of the alloy, it can be concluded that precipitation strengthening has the greatest influence on the strength improvement of the alloy system after cold rolling and aging treatments. The dispersion of the Cr atoms on a nanometer scale has the greatest effect on the strength of the alloy. Dislocation strengthening and grain boundary strengthening in the alloy also contribute greatly to the improvement of the alloy strength, which is because the grain of the alloy is refined after large deformation, and a large number of dislocations are generated in the grain, resulting in a large deformation resistance during the alloy deformation process. Since Cr atoms are almost completely precipitated in the alloy, Ni atoms have little effect on the lattice distortion of the Cu matrix, so the solution strengthening in the alloy has little contribution to the improvement of the alloy’s strength.

The electrical conductivity of the alloy is affected by the internal defects of the alloy. Solute atoms, dislocations, precipitates, and grain boundaries have adverse effects on the electrical conductivity of the alloy by scattering electrons. Without considering the temperature effect, Mattiessen’s law [39] can be used to explain the resistance of the aging Cu-Ni-Cr alloys.

(14) $$\rho =\sum_{i}\rho_{i}=\rho_{0}+\rho_{GB}+\rho_{d}+\rho_{p}+\rho_{ss}$$

where ρ0 is the resistivity of the annealed pure copper at room temperature and $\rho_{GB}$, $\rho_{d}$, $\rho_{p}$, and $\rho_{ss}$ are the resistivity of the alloy due to grain boundaries, dislocations, precipitates, and solute atoms, respectively.

In the aging Cu-Ni-Cr alloy, the main factors affecting the conductivity of the alloy include solid solution atoms, the dislocation density and grain boundary density, and precipitates. The almost complete solution of Ni in the copper matrix causes lattice distortion, which increases the scattering ability of electrons and is the most important factor affecting the electrical conductivity of the alloy. Grain boundaries and dislocations in the matrix have a certain scattering stress on electrons, which increases the resistivity of the alloy. The precipitates of the alloy effectively reduce the conductive volume, which leads to a decrease in the conductivity of the alloy. The precipitates of the nano-scale Cr in the BCC structure are in a semi-coherent relationship with the matrix, which produces local lattice distortion. The introduction of the phase interface and the local lattice distortion caused by the precipitates reduce the conductivity of the alloy. For Cu-Ni-Cr alloys with rolling strains of 3.2 and 4.3, due to the similar solid solubility of the Ni elements in the alloys, the influence of the solid solution atoms on the conductivity is similar. Their grain boundary density and dislocation density are different, but the difference is too small to make a significant difference in macroscopic conductivity [29]. The number of precipitates produced by the alloys under the two stress variables are the same, but the distribution states are different. When the rolling strain is 4.3, the number of precipitates produced by the alloys under the two stress variables is the same, but the distribution state is different. 

When the rolling strain reaches 4.3, due to the increase of the alloy reduction rate, the deformation energy increases and nucleation points increase, resulting in more Cr precipitates in the alloy and a smaller size. The study of Huang et al. [40] showed that when the distance between the nano-scale precipitates in copper was smaller than the travel distance of free electrons (42 nm), the nano-scale precipitates significantly affected the conductivity of the alloy. In this study, when the rolling strain reaches 4.3, many nano-scale Cr precipitates are dispersed, and the gap of many nano-scale Cr precipitates is less than 42 nm, as shown in Figure 12c. The nano-scale precipitates with small sizes have a greater impact on the conductivity of the alloy.

In conclusion, under different reduction rates, factors such as the solution, grain boundary, and dislocation have little influence on conductivity. However, the large deformation further reduces the spacing of the nano-scale precipitates, resulting in an increase in the number of nano-scale precipitates whose spacing is smaller than the travel distance of the free electrons, which is the main reason for the decrease in conductivity.

## 5. Conclusions

1. The conductivity and strength of the Cu-1.16Ni-0.36Cr alloy are greatly improved after the thermo-mechanical treatment process. With an increase of the strain variable, these decrease slightly. When the rolling strain reaches 3.2, the strength reaches 512 MPa and the conductivity reaches 45.5% IACS. At a rolling strain of 4.3, the strength further increases to 536.1 MPa and the conductivity decreases to 41.9% IACS.
2. After thermo-mechanical treatment, the grain size of the Cu-1.16Ni-0.36Cr alloy decreased significantly. As the rolling strain increased, more deformation energy was accumulated within the alloy, leading to an increase in the degree of recrystallization after the aging treatment. During this process, the grain size initially decreased but then started to increase. Specifically, when the rolling strain increased from 3.2 to 4.3, the recrystallization degree of the alloy increased from 7.1% to 20.2%. However, contrary to expectations, the grain size of the alloy also increased slightly, from 3.42 μm to 3.88 μm.
3. Cr exists in the form of nano-scale granular precipitates in the alloy. There are two kinds of particles at the same time, which are little nano-sized precipitates with the size of more than 100 nm and many nano-scale precipitates with the average size of about 10 nm. The nano-scale Cr precipitates showed a semi-coherent relationship with the matrix. As the deformation amount increases during cold rolling, more deformation energy is accumulated within the alloy. This accumulation of energy promotes the dispersion and precipitation of the nano-scale precipitates during the subsequent aging treatment.
4. Quantitative calculations reveal that the primary strengthening mechanisms of the alloy include grain boundary strengthening induced by significant deformation and precipitation strengthening caused by nanometer-sized Cr precipitates. When the rolling strain reaches 3.2, the grain boundary strengthening contributes to 30.8% of the overall strength, while the precipitation strengthening from the nanometer-sized Cr precipitates contributes 28.3%. With an increase in the rolling deformation, the dislocation density decreases, and the volume fraction of precipitates increases after the aging treatment. When the rolling strain reaches 4.3, the contribution of the two strengthening mechanisms becomes 21.5% and 33.0%, respectively.

## Data Availability

Data will be made available on request.

## References

[1] Zhou F., Zhang Y., Lu L., Song K., Gao H. (2022). Effects of a thermal-ultrasonic stress relaxation process on the residual stress, mechanical properties and microstructures of C19400 copper alloy strips. Mater. Sci. Eng. A.

[2] Shan L., Yang L., Wang Y. (2022). Improving the high temperature mechanical performance of Cu–Cr alloy induced by residual nano-sized Cr precipitates. Mater. Sci. Eng. A.

[3] Wang W., Kang H., Chen Z., Chen Z., Zou C., Li R., Yin G., Wang T. (2016). Effects of Cr and Zr additions on microstructure and properties of Cu-Ni-Si alloys. Mater. Sci. Eng. A.

[4] Li X., Li Z., Cheng X., Sun W., Zheng Y., Yu Q., Wang C., Wang Q., Dong C. (2019). Precipitation evolution in Cu [Ni3Cr1] spinodal alloys under mismatch control. Mater. Chem. Phys..

[5] Qiu C., Hu B., Zhou J., Wu P., Liu Y., Wang C., Du Y. (2020). The phase equilibria of the Cu–Cr–Ni and Cu–Cr–Ag systems: Experimental investigation and thermodynamic modeling. Calphad.

[6] Hernandez-Santiago F., Lopez-Hirata V.M., Saucedo-Muñoz M.L. (2010). Coarsening Process of Decomposed Phases in Cu-Ni-Cr Alloys. Mater. Sci. Forum.

[7] Xu G., Liu Y., Lei F., Sheng G., Kang Z. (2015). Diffusion behavior and atomic mobilities for fcc Cu–Cr–Ni alloys. J. Alloys Compd..

[8] Hernandez-Santiago F., Lopez-Hirata V., Dorantes-Rosales H.J., Saucedo-Muñoz M.L., Gonzalez-Velazquez J.L., Paniagua-Mercado A.M. (2008). Ostwald ripening of decomposed phases in Cu–Ni–Cr alloys. J. Alloys Compd..

[9] Trivedi V., Battabyal M., Murty B.S., Gopalan R. (2022). Interfacial thermoelectric and mechanical properties of indigenously prepared Ni–Cr–Cu/Co4Sb12 skutterudite thermoelectric joints. Ceram. Int..

[10] Wang G., Liu H., Song K., Zhou Y., Cheng C., Guo H., Guo Y., Tian J. (2022). Aging process and strengthening mechanism of Cu–Cr–Ni alloy with superior stress relaxation resistance. J. Mater. Res. Technol..

[11] Fu H., Xu S., Li W., Xie J., Zhao H., Pan Z. (2017). Effect of rolling and aging processes on microstructure and properties of Cu-Cr-Zr alloy. Mater. Sci. Eng. A.

[12] Tu Y., Wang W., Liu X., Feng Q. (2022). The compositional homogenization control of Cu-Ti alloys prepared by accumulative roll bonding-deformation diffusion process. J. Alloys Compd..

[13] Sun X., Jie J., Wang T., Li T. (2021). Effect of two-step cryorolling and aging on mechanical and electrical properties of a Cu–Cr–Ni–Si alloy for lead frames applications. Mater. Sci. Eng. A.

[14] Wu Y.H., Song L.P., Yin Z.M., Dai J.Y., Zhang J. (2007). Influence of Thermomechanical Treatment on Microstructure and Mechanical Properties of Cu-Ni-Cr Alloy. Min. Metall. Eng..

[15] (2010). Metallic Materials—Tensile Testing—Part 1: Method of Test at Room Temperature.

[16] Williamson G.K., Hall W.H. (1953). X-ray line broadening from filed aluminum and wolfram. Acta Metall..

[17] Williamson G.K., Smallman R.E. (1956). Dislocation densities in some annealed and cold-worked metals from measurements on the X-ray debye-scherrer spectrum. Philos. Mag..

[18] Zhao Y., Liao X., Jin Z., Valiev R., Zhu Y. (2004). Microstructures and mechanical properties of ultrafine grained 7075 Al alloy processed by ECAP and their evolutions during annealing. Acta Mater..

[19] Leffers T., Ray R.K. (2009). The brass-type texture and its deviation from the copper-type texture. Prog. Mater. Sci..

[20] Chang L., Wen S., Li S., Zhu X., Shang X. (2015). Strain softening during tension in cold drawn Cu–Ag alloys. Mater. Charact..

[21] Gil Sevillano J., Van Houtte P., Aernoudt E. (1977). The contribution of macroscopic shear bands to the rolling texture of FCC metals. Scr. Metall..

[22] Wang Y., Huang H.-Y., Xie J.-X. (2011). Texture evolution and flow stress of columnar-grained polycrystalline copper during intense plastic deformation process at room temperature. Mater. Sci. Eng. A.

[23] Xie M., Huang W., Chen H., Gong L., Xie W., Wang H., Yang B. (2021). Microstructural evolution and strengthening mechanisms in cold-rolled Cu–Ag alloys. J. Alloys Compd..

[24] Liu J.B., Meng L. (2008). Phase orientation, interface structure, and properties of aged Cu-6 wt.% Ag. J. Mater. Sci..

[25] Avrami M. (1939). Kinetics of Phase Change. I General Theory. J. Chem. Phys..

[26] Raeisinia B., Poole W., Lloyd D. (2006). Examination of precipitation in the aluminum alloy AA6111 using electrical resistivity measurements. Mater. Sci. Eng. A.

[27] Davis J.R. (2001). ASM Specialty Handbook: Copper and Copper Alloys.

[28] Wu Y., Li Y., Lu J., Tan S., Jiang F., Sun J. (2019). Correlations between microstructures and properties of Cu-Ni-Si-Cr alloy. Mater. Sci. Eng. A.

[29] Labusch R. (1970). A Statistical Theory of Solid Solution Hardening. Phys. Status Solidi B.

[30] Staker M., Holt D. (1972). The dislocation cell size and dislocation density in copper deformed at temperatures between 25 and 700°C. Acta Metall..

[31] Jiang L., Fu H., Wang C., Li W., Xie J. (2020). Enhanced Mechanical and Electrical Properties of a Cu-Ni-Si Alloy by Thermo-mechanical Processing. Metall. Mater. Trans. A.

[32] Liao W., Yang H., Yi C., Zheng J. (2022). Effect and mechanism of cold rolling and aging process on microstructure and properties of columnar grain C70250 copper alloy. Mater. Sci. Eng. A.

[33] Zhao Z., Xiao Z., Li Z., Qiu W., Jiang H., Lei Q., Liu Z., Jiang Y., Zhang S. (2019). Microstructure and properties of a Cu-Ni-Si-Co-Cr alloy with high strength and high conductivity. Mater. Sci. Eng. A.

[34] Petch N.J. (1953). The cleavage strength of polycrystals. J. Iron Steel Inst..

[35] Xu S., Fu H., Wang Y., Xie J. (2018). Effect of Ag addition on the microstructure and mechanical properties of Cu-Cr alloy. Mater. Sci. Eng. A.

[36] Huang A., Wang Y., Wang M., Song L., Li Y., Gao L., Huang C., Zhu Y. (2019). Optimizing the strength, ductility and electrical conductivity of a Cu-Cr-Zr alloy by rotary swaging and aging treatment. Mater. Sci. Eng. A.

[37] Argon A.S., Orowan E. (1964). Plastic deformation in mgo single crystals. Philos. Mag..

[38] Peng H., Xie W., Chen H., Wang H., Yang B. (2021). Effect of micro-alloying element Ti on mechanical properties of Cu–Cr alloy. J. Alloys Compd..

[39] Liao W., Liu X., Yang Y., Wang S., Du M. (2019). Effect of cold rolling reduction rate on mechanical properties and electrical conductivity of Cu–Ni–Si alloy prepared by temperature controlled mold continuous casting. Mater. Sci. Eng. A.

[40] Huang K. (2014). Solid State Physics.

